# Endoscopic management is the preferred “treatment” modality for grade III vesicoureteric reflux with breakthrough infections in a young girl

**DOI:** 10.4103/0970-1591.44250

**Published:** 2008

**Authors:** R. B. Nerli

**Affiliations:** Department of Urology, KLES Kidney Foundation, Nehrunagar, Belgaum-590 010, India

**Keywords:** Endoscopy, open surgery, uropathy, vesicoureteral reflux

## Abstract

Endoscopic subureteric injection of tissue-augmenting substances has become an alternative to long-term antibiotic prophylaxis and open surgery, in the treatment of children with vesicoureteric reflux (VUR). Successful elimination of reflux in about 80% of patients after a single injection (and in 90% after a repeat) has been achieved using non-degradable substances. young girl with grade III VUR and breakthrough infections would definitely need to undergo antireflux procedure. Endoscopic treatment would be an ideal procedure as it is a one-day surgery, with over 80% success rate, low morbidity and no long-term complications. Moreover, this form of surgery is appealing to, as well as the choice of the majority of parents.

## INTRODUCTION

Vesicoureteral reflux (VUR) is the most common uropathy in children (0.4–1.8% of pediatric population); nevertheless, its optimal management remains controversial. Until the 1980s, treatment guidelines recommended antibiotic prophylaxis (AP) as “therapy” for mild-grade reflux (I-II). Antibiotic prophylaxis was also indicated as initial therapy for grades III-IV. Open surgery was recommended for patient with high-grade (IV-V) or persistent (any grade) reflux.[1]

Endoscopic treatment (ET) of reflux by means of subureteral injection of bulking materials was first described by Matouschek in 1981 and further developed by Puri and O'Donnel.[2–4] Since then, ET has gained popularity and has proved successful in a high percentage of cases. Endoscopic treatment is now considered a valid alternative to open surgery and antibiotic prophylaxis.[5] Endoscopic treatment using dextranomer/hyaluronic acid copolymer (Deflux) or other bulking agents approved by the US Food and Drug Administration is indicated when conservative treatments have failed:

- Lower grades of reflux (grades I to III); or
- Recurrent, poorly controlled febrile urinary tract infections (UTI); or
- Persistent reflux in post-pubertal female members; or
- Deterioration of renal parameters regardless of reflux severity; or
- Children who have had a previously unsuccessful ureteral reimplantation; or
- Children who have stopped taking medication as a result of drug intolerance or parental non-compliance.

## STUDY CASE

A young girl with Grade III VUR and breakthrough infections is an ideal case for ET. Since she has had breakthrough infections on conservative treatment with antibiotic prophylaxis, she would need to undergo some form of antireflux procedure. Moreover she being a young girl, she would be soon entering post-pubertal phase into reproductive age group with a persistent reflux. Endoscopic treatment would be the right choice of antireflux procedure as it is a one-day surgery, with over 80% success rate, low morbidity and no long-term complications. Endoscopic treatment of VUR offers the advantage of enabling treatment of the underlying anatomical defect while avoiding the morbidity of open surgery.[6]

## DISCUSSION

The indications for correction of reflux remain unchanged regardless of whether reflux is corrected by open surgery, endoscopy or laparoscopy. Breakthrough febrile UTI or pyelonephritis during antibiotic prophylaxis are generally considered an indication for termination of watchful waiting and correcting the reflux. Capozza and Caione[6] opine that, the advent of ET has changed the algorithm of reflux management in children. Endoscopic treatment is minimally invasive, can be performed as a one-day surgery (or even as an outpatient procedure) and has very low morbidity. Currently used injectable materials are safe and ensure long-term permanence at the site of injection. Capozza and Caione,[6] on the basis of the success rate of ET, proposed that endoscopic treatment should be the first-line option for most cases of VUR, a useful alternative to antibiotic prophylaxis in low-grade reflux and to open surgery in high-grade reflux.

Elder *et al.*,[7] performed a meta-analysis of the existing literature pertaining to endoscopic treatment to allow comparison with reports of open surgical correction. The database included 5527 patients and 8101 renal units. Following one treatment the reflux resolution rate for Grades I and II reflux was 78.5%, Grade III 72%, Grade IV 63% and Grade V 51%. If the first injection was unsuccessful, the second treatment had a success rate of 68%, and the third treatment 34%. The aggregate success rate with one or more injections was 85%. After endoscopic treatment with variable follow-up, pyelonephritis developed in 0.75% of patients and cystitis in 6%. There were few reports of renal scarring following treatment. They concluded that endoscopic treatment provides a high rate of success in children with reflux. Reports to date have not indicated any additional difficulty with open surgery after endoscopic correction with Deflux,[8,9] although some have experienced difficulty with other substances. At open surgery the injected material either is not seen at all or is found well encapsulated but in a wrong plane or wrong location inside or outside the bladder. The material is easily removed en bloc and the open reimplant procedure carried out without difficulty.

Probably no other topic in pediatric uro-nephrology has been so hotly debated as the surgical management of VUR.[10] It is clear that in general, all open surgical techniques have a high success rate exceeding 95% in the hands of qualified pediatric urologists.[11] Trials designed to assess the incremental benefit of open surgery over antibiotics alone have been conducted[12–14] and these studies have not shown any additional benefit of open surgery except for a reduction in risk of febrile urinary tract infections. Nijman[15] in a famous and controversial editorial commentary, concluded that these results further support the concept that renal damage has already occurred at a very early stage and that, maybe, the only reason for open surgical treatment in these children is to give the physician the feeling that everything possible has been done to prevent further UTI and renal damage.

Complications following open surgical reimplantation include persistent reflux, contralateral reflux and ureteric obstruction. In the AUA Guidelines report, open surgical correction was associated with persistent reflux in 2% and ureteral obstruction in 2%.[16] Higher risk of progressive hydronephrosis, UTI, hypertension, renal failure, spontaneous abortion and premature birth during pregnancy was reported in some patients undergoing ureteral reimplantation in childhood.[17,18] It is therefore important to be aware of these late complications, and female patients who have undergone antireflux surgery require close monitoring during pregnancy.

Some studies have been performed to assess the cost and outcome of ET for VUR compared with antibiotics and open surgery.[19] Conclusions of these studies are that ET for VUR appears to be cost-effective when compared with open surgery. Parents of 100 children with Grade III reflux (38 boys and 62 girls, mean age four years, range 1–15) were provided with detailed information about the three treatment options: antibiotic prophylaxis, open surgery and ET. Most parents (80%) preferred ET rather than antibiotic prophylaxis (5%) or open surgery (2%), 13% could not decide among the three options and ET was recommended.[20]

## CONCLUSIONS

A young girl with persistent Grade III VUR, and recurrent urinary tract infection in spite of antibiotic prophylaxis would be best treated by ET as ET can be easily performed as a one-day surgery, is associated with low morbidity and over 80% success rate. The more recent studies report success rates approaching 90% after one injection of Deflux for low-grade primary reflux.[21]
